# Impact of Substance Use Navigators on Initiation of Treatment for Substance Use Disorders and 30-Day Unplanned Readmission

**DOI:** 10.1007/s11606-025-09902-y

**Published:** 2025-10-20

**Authors:** Arianna Campbell, Allison D. Rosen, Steven J. Shoptaw, Andrew A. Herring, David Jay, Aimee Moulin, Juliet LaMers, Elizabeth A. Samuels, Lindsay Gietzen

**Affiliations:** 1 Department of Health Sciences, University of the Pacific, Sacramento, CA USA; 2 The Bridge Center, Public Health Institute, Oakland, CA USA; 3 Department of Family Medicine, David Geffen School of Medicine, University of California Los Angeles, Los Angeles, CA USA; 4 Department of Emergency Medicine, Highland General Hospital – Alameda Health System, Oakland, CA USA; 5 Department of Emergency Medicine, University of California San Francisco, San Francisco, CA USA; 6 Department of Emergency Medicine, David Geffen School of Medicine, University of California Los Angeles, Los Angeles, CA USA; 7 Department of Psychiatry and Biobehavioral Sciences, David Geffen School of Medicine, University of California Los Angeles, Los Angeles, CA USA; 8 Department of Emergency Medicine, University of California, Davis, Sacramento, CA USA; 9 Department of Population Health, Marshall Medical Center, Placerville, CA USA; 10 Department of Emergency Medicine, VA Northern California Healthcare System, Mather, CA USA; 11 Marshall CARES, Marshall Medical Center, Placerville, CA USA

**Keywords:** hospital, addiction, navigator, rural, MOUD, MAUD

## Abstract

**Background:**

The prevalence of substance use disorders (SUDs) is high among hospitalized patients, yet adoption of evidence-based practices to address SUDs in this population is limited. Substance use navigators (SUNs) are a promising intervention for improving patient outcomes.

**Objective:**

This study aimed to quantify the impact of SUN consultations on medications for opioid use disorder (MOUD) and medications for alcohol use disorder (MAUD) initiation and 30-day unplanned readmissions in hospitalized patients with SUDs.

**Design:**

A retrospective observational cohort study at a rural hospital in Northern California following adoption of the CA Bridge model of using SUNs to provide low-threshold, evidence-based treatment for SUDs. Patient data were extracted from electronic medical records.

**Patients:**

Hospitalized patients with a primary or secondary diagnosis of alcohol use disorder or opioid use disorder in 2023.

**Main Measures:**

The two outcomes of interest were MOUD and MAUD initiation and 30-day unplanned readmission. Propensity score matching was used to estimate risk ratios comparing outcomes for patients who did vs. did not receive SUN consultation.

**Key Results:**

Among 781 patients, 26.6% received a SUN consultation and the most common primary diagnosis was sepsis (9%). MOUD and MAUD were initiated among 15.7% of patients who had a SUN consultation and 7% who did not, and the 30-day unplanned readmission rate was 4% for patients who had a SUN consultation and 10.9% for those who did not. In the propensity score matched analysis, having a SUN consultation was associated with a twofold increase in MOUD/MAUD initiation (RR 2.29, 95%CI 1.27–4.11) and a 64% reduction in risk of 30-day unplanned readmission (RR 0.36, 95%CI 0.17–0.80).

**Conclusions:**

Adoption of SUNs may be an effective strategy for improving quality of care for patients with SUDs and reducing costs associated with unplanned readmissions at community-based hospitals without addiction medicine consult services.

## INTRODUCTION

At least 12% of hospitalized patients have a substance use disorder (SUD), yet few of these patients receive evidence-based treatment for their SUD during their hospitalization.^1–3^ Even after the Society of Hospital Medicine’s 2022 issue of guidelines recommending initiation of FDA-approved medications and linkage to ongoing care post discharge, adoption of evidence-based practices for the treatment of SUDs in hospitals has been slow and inconsistent.^1,3,4^ Consequently, patients with SUDs experience higher than usual rates of patient-directed discharges, readmissions, and mortality, particularly when their SUD is not effectively addressed.^5,6^ This omission of care for SUDs during hospital stays is especially grave for individuals with opioid use disorder (OUD), who face a nearly 8% mortality rate within the year following hospital discharge.^7^

Hospitals represent a critical access point for the initiation of SUD treatment, particularly for individuals excluded from treatment due to disparities in insurance coverage, transportation, identification requirements, and other poverty and systemic issues. ^8^ Addressing SUDs and establishing standards for hospital-based treatment can both improve quality of care for patients and reduce costs for hospitals.^1^ Decreasing 30-day unplanned readmissions is an important goal for many hospitals, as the Center for Medicare & Medicaid Services’ (CMS) Hospital Readmissions Reduction Program (HRRP) reduces reimbursements for hospitals with excess 30-day unplanned readmissions among patients with diagnoses such as chronic obstructive pulmonary disease (COPD), acute myocardial infarction, and hip and knee joint replacements. Although SUDs are not one of the diagnoses assessed by the HRRP, 30-day unplanned readmission rates are high for this group, and comorbid SUDs likely contribute to readmissions among patients with other primary diagnoses.

The use of substance use navigators (SUNs) is a promising strategy for both increasing initiation of evidence-based treatment during hospitalization and reducing 30-day unplanned readmissions for patients with SUDs.^3,9,10^ SUNs are trained in harm reduction, motivational interviewing, trauma-informed care, may have lived experience with substance use, and are often trained alcohol and drug counselors; they work with clinical providers to advocate for the use of medications for opioid use disorder (MOUD) and medications for alcohol use disorder (MAUD), and link patients to outpatient services post discharge.^8,9^

At Marshall Medical Center (Marshall), a substance use navigator (SUN) has been serving as part of the Emergency Department (ED) and hospital care team since 2019. The SUN engages in care by receiving requests from physicians and APPs, nurses, social workers, or through monitoring ED and hospital admissions through the electronic medical record (EMR) system. SUN consults are tracked through consults that are placed through the EMR by any of these members of the care team. The placement of the consult generates a list that is reviewed by the SUN at the initiation of their shift and throughout their shift. The SUN will triage the consult requests and if consult volume exceeds capacity of the SUN, the SUN prioritizes patients with any SUD in acute crisis, and then patients with OUD. Patients in the ED are prioritized, then hospitalized patients are seen next, and then patients with alcohol use disorder (AUD). The SUN will also respond with urgency to a patient who is determined to be at risk of self-directed discharge from the hospital.

Marshall initiated its SUN program after receiving funding through a grant provided by The California Department of Healthcare Services State Opioid Response grant funded through the CA Bridge Program.^10^ The SUN is currently funded by Marshall’s Emergency Department (ED) with intermittent grant funding. There is currently an effort to capture reimbursement through community health worker (CHW) billing and Medicare navigator reimbursement.

The SUN at Marshall is trained in a “medication first” approach^11^, with training facilitated by the CA Bridge program.^10^ The SUN is also a certified drug and alcohol counselor trained in Screening, Brief Intervention and Referral to Treatment (SBIRT)^12^ as well as Motivational Interviewing (MI)^13^ through a lens of connecting to medications.^11^ The SUN connects patients to “low barrier” clinics in the area who continue medications. With a focus on the support of initiating MOUD and MAUD, the SUN works closely with the medical team to advocate for medications and provide resources to support the initiation of medications. Prescribers in the hospital setting also received training through technical assistance provided by the CA Bridge program as well as by the local clinical champion.

This study aimed to assess the impact of the SUN consult on initiation of MOUD/MAUD and rates of 30-day unplanned readmission among patients hospitalized with AUD or OUD at Marshall, a rural hospital in Northern California.

## METHODS

### Study Design and Intervention

This study was conducted as part of a quality improvement initiative at Marshall Medical Center (Marshall), a rural hospital in Northern California. In 2017, as part of the CA Bridge initiative, Marshall implemented a program to provide evidence-based, low-threshold treatment for patients with SUDs. This included hiring a SUN to facilitate treatment and linkage to outpatient care as well as providing MOUD and MAUD. MOUD is an evidence-based intervention that significantly reduces mortality risk and increases retention in treatment.^4,5^ MAUD is effective in decreasing craving and heavy drinking days and is often overlooked in hospitalized patients with AUD.^2,14^

Providers at Marshall primarily prescribe buprenorphine for medication for opioid use disorder (MOUD) and naltrexone for medication for alcohol use disorder (MAUD). While larger studies have evaluated the impact of implementing the CA Bridge model in emergency departments, it has not been evaluated in inpatient settings.^10,15,16^ The SUN at Marshall is based in the ED but routinely sees patients who are admitted to the hospital as part of their daily work. Since the number of SUN referrals often outnumbers the number of consults that can be completed in one day, the SUN will prioritize any patient in acute crisis, then ED patients followed by admitted patients due to length of time available for contact.

Marshall is a 111-bed independent, non-profit, single hospital in its own healthcare system in the foothills of Northern California. Census data indicates a population of 10,730 in Placerville, the city where Marshall Hospital is located. Placerville has 15.7% of residents living at or below the poverty line, which is 1.3 times the poverty rate in California.^17^ Marshall is one of only two hospitals in El Dorado County. With the closest hospital 23 miles away, CMS HRRP offers data that demonstrates that readmission of Marshall patients to outside hospitals is < 1%.^18^

There were 32 overdose deaths in El Dorado County in 2023, most in Placerville.^19^ Overdoses peaked in 2021.^20^ Buprenorphine prescribing rates significantly increased from 2019 through 2023 during the time of CA Bridge implementation and consistent with statewide increases in ED and hospital-initiated buprenorphine.^16^

Data were obtained from hospitalized patients admitted to Marshall in calendar year 2023 through Epic Electronic Medical Record (EMR). Included in the study were those over the age of 18 with a primary or secondary diagnostic ICD-10 code in defined categories of AUD (F10) or OUD (F11, F19). Patients were excluded if they were ineligible for readmission (i.e., transferred to another facility or died during the hospitalization) and following the CMS exclusions for readmission definition, which notably excludes patient-directed discharges.^18^ This study was reviewed by the University of the Pacific Institutional Review Board (IRB) and considered to be quality improvement and not human subjects research.

### Main Measures

The primary exposure of interest was receipt of a consultation with the SUN during the hospitalization. The two outcomes of interest were MOUD/MAUD initiation during the hospital stay and 30-day unplanned readmission. MOUD was defined as FDA-approved medications for OUD (buprenorphine or methadone) and/or MAUD was defined as medications for AUD (naltrexone or acamprosate). Other medications for AUD were not included as they had more broad clinical indications or lack of utilization in the inpatient setting. Readmissions were defined per CMS guidelines under the Hospital Readmissions Reduction Program (HRRP) as standardized unplanned readmissions to the same hospital within 30 days of the initial admission. ^18^ Covariates of interest included age in years, sex, race or ethnicity, health insurance, length of stay in days, and discharge disposition.

### Analytic Strategy

Propensity score matching was used to estimate average treatment effects for the treated (ATT) for each exposure-outcome relationship of interest. We used 1:1 nearest neighbor matching with caliper width of 0.2 of the pooled standard deviation of the logit of the propensity score and without replacement.^21^ Propensity scores were calculated using variables associated with both exposure and outcome and the outcome only; variables were chosen based on literature review. Propensity scores used to estimate the ATT for the effect of receiving a SUN consult on MOUD/MAUD initiation included age, sex, race or ethnicity, whether the patient was a Medicaid beneficiary, whether the patient had an OUD diagnosis, and length of stay in days. Propensity scores used to estimate the ATT for the effect of receiving a SUN consult on 30-day unplanned readmission included all aforementioned factors as well as discharge disposition, defined as being discharged home under self-care or discharged with care such as a home health service or to a facility; this categorization was chosen based on findings from another study showing that the increased risk of 30-day unplanned readmission for patients with substance use disorders only held for patients who were discharged home under self-care.^6^

Balance between groups was checked for each covariate in the propensity score models and an absolute standardized mean difference ≤ 0.1 was considered balanced.^21^ Balance diagnostics revealed that defining length of stay in quartiles yielded the best balance between the exposed and unexposed individuals. Risk ratios with robust 95% confidence intervals accounting for dependence induced from matching were calculated for each propensity score matched sample.

All analyses were conducted in R version 4.2.1 using the MatchIt package, and a *p*-value less than 0.05 was considered statistically significant.^22^ This study was approved by the University of the Pacific Institutional Review Board (IRB) and all data was de-identified. SQUIRE reporting guidelines were followed. ^23^

## RESULTS

**Figure 1:**
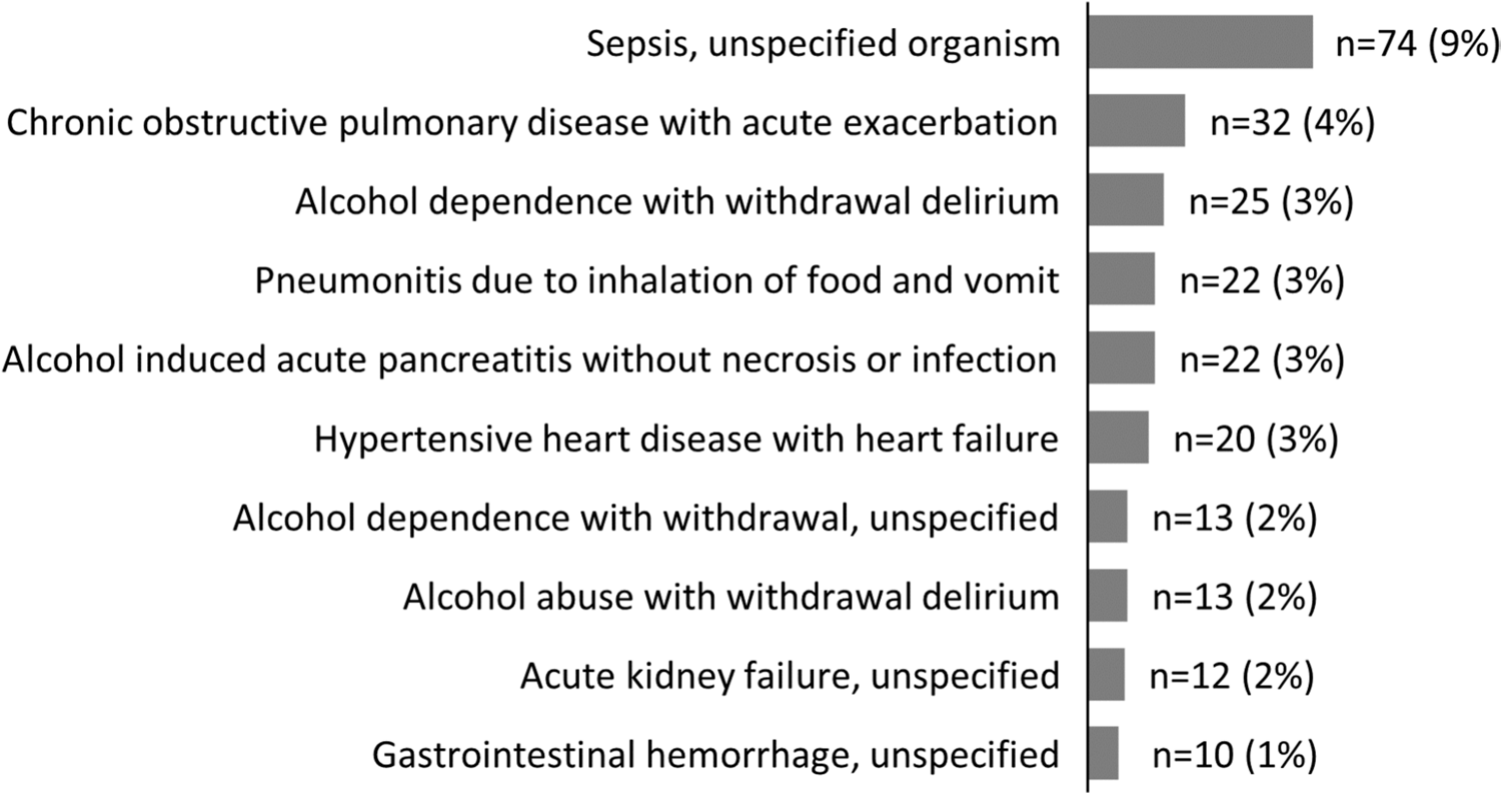
Top 10 primary diagnoses at discharge (*n* = 781).

Among 903 patients admitted to Marshall with AUD or OUD in 2023, 781 were eligible for inclusion in this analysis. Among these patients, 208 (26.6%) received a SUN consult and 573 (73.4%) did not. Additionally, 62 (7.9%) patients had both AUD and OUD, 376 (48.1%) had AUD only, and 343 (43.9%) had OUD only. The most common primary diagnoses at discharge were sepsis (unspecified organism) (*n* = 74, 9%), followed by chronic obstructive pulmonary disease with acute exacerbation (*n* = 32, 4%), and alcohol dependence with withdrawal delirium (*n* = 25, 4%) (Fig. 1).

Propensity score matching for the purpose of calculating the ATT for the effect of receiving a SUN consult on MOUD/MAUD initiation yielded matches for 201 (96.6%) of the 208 patients who received a SUN consult, yielding a matched sample size of 402. These patients had an average age of 54.3 years; 147 (36.6%) were female, 349 (86.6%) identified as non-Hispanic white, 201 (50.0%) were Medicaid beneficiaries, and 136 (33.8%) were diagnosed with OUD. Absolute standardized mean differences show adequate balance in the matched sample, as compared to the full sample before matching (Table 1).

**Table 1:** Baseline Characteristics of Propensity Score Matched Sample for Analysis of MOUD/MAUD Initiation

		**SUN consult**			
	**Total** **(*n* = 402)**	**Yes** **(*n* = 201)**	**No** **(*n* = 201)**	**Full sample**	**Matched sample**
	***n* (%)**	***n* (%)**	***n* (%)**	**ASMD**	**ASMD**
**Age, mean (SD)**	54.3 (14.7)	54.0 (14.0)	54.6 (15.4)	0.84	0.04
**Female sex**	147 (36.6)	78 (38.8)	69 (34.3)	0.21	0.09
**Race or ethnicity**					
White	349 (86.8)	175 (87.1)	174 (86.6)	0.14	0.01
Hispanic or Latine	28 (7.0)	14 (7.0)	14 (7.0)	0.14	0.00
Other	25 (6.2)	12 (6.0)	13 (6.5)	0.04	0.02
**Medicaid insurance**	201 (50.0)	101 (50.2)	100 (49.8)	0.48	0.01
**OUD diagnosis**	136 (33.8)	72 (35.8)	64 (31.8)	0.49	0.08
**Length of stay**^**1**^					
1–2 days	98 (24.4)	46 (22.9)	52 (25.9)	0.31	0.07
3 days	93 (23.1)	43 (21.4)	50 (24.9)	0.03	0.08
4–5 days	92 (22.9)	47 (23.4)	45 (22.4)	0.04	0.02
6 or more days	119 (29.6)	65 (32.3)	54 (26.9)	0.27	0.10

*MOUD*, medication for opioid use disorder; *MAUD*, medication for alcohol use disorder; *SD*, standard deviation; *OUD*, opioid use disorder; *ASMD*, absolute standardized mean difference

^1^Categorized in quartiles of full sample

Among patients who received a SUN consult, 15.9% initiated MOUD/MAUD compared to 7% of those who did not receive a SUN consult. Patients who received a SUN consult were over twice as likely to initiate MOUD/MAUD compared to those who did not receive a SUN consult (aRR 2.29, 95%CI 1.27–4.11) (Table 3).

Propensity score matching for the purpose of calculating the ATT for the effect of receiving a SUN consult on 30-day unplanned readmission yielded matches for 202 (97.1%) of the 208 patients who received a SUN consult, yielding a matched sample size of 404. These patients had an average age of 54.2 years; 148 (36.6%) identified as female, 347 (85.9%) identified as non-Hispanic white, 205 (50.7%), were Medicaid beneficiaries, 141 (34.9%) were diagnosed with OUD, and 78.5% were discharged home under self-care. Absolute standardized mean differences show adequate balance in the matched sample, as compared to the full sample before matching (Table 2).

**Table 2:** Baseline Characteristics of Propensity Score Matched Sample for Analysis of 30-Day Unplanned Readmissions

		**SUN consult**		**Full sample**	**Matched sample**
	**Total** **(*n *= 404)**	**Yes** **(*n* = 202)**	**No** **(*n* = 202)**		
	**n (%)**	***n* (%)**	***n* (%)**	**ASMD**	**ASMD**
**Age, mean (SD)**	54.2 (14.9)	53.9 (14.0)	54.4 (15.8)	0.84	0.04
**Female sex**	148 (36.6)	77 (38.1)	71 (35.1)	0.21	0.06
**Race or ethnicity**					
White	347 (85.9)	173 (85.6)	174 (86.1)	0.14	0.01
Hispanic or Latine	31 (7.7)	16 (7.9)	15 (7.4)	0.14	0.02
Other	26 (6.4)	13 (6.4)	13 (6.4)	0.04	0.00
**Medicaid insurance**	205 (50.7)	101 (50.0)	104 (51.5)	0.48	0.03
**OUD diagnosis**	141 (34.9)	72 (35.6)	69 (34.2)	0.49	0.03
**Length of stay**^**1**^					
1–2 days	98 (24.3)	46 (22.8)	52 (25.7)	0.31	0.07
3 days	91 (22.5)	43 (21.3)	48 (23.8)	0.03	0.06
4–5 days	93 (23.0)	49 (24.3)	44 (21.8)	0.04	0.06
6 or more days	122 (30.2)	64 (31.7)	58 (28.7)	0.27	0.06
**Discharged to home**	317 (78.5)	157 (77.7)	160 (79.2)	0.18	0.04

*SD*, standard deviation; *OUD*, opioid use disorder; *ASMD*, absolute standardized mean difference

^1^Categorized in quartiles of full sample

Among patients who received a SUN consult, 4.0% had a 30-day readmission compared to 10.9% of those who did not receive a SUN consult. Patients who had a SUN consult had a 64% reduction in risk of 30-day readmission compared to patients who did not have a SUN consult (aRR 0.36, 95%CI 0.17–0.80) (Table 3).

**Table 3:** Association of SUN Consults with MOUD/MAUD Initiation and 30-Day Unplanned Readmission for Inpatients with AUD or OUD

		***n* (%)**	***n* (%)**	**RR (95% CI)**
		**Received MOUD/MAUD**		
		**Yes**	**No**	
**SUN consult**	**Yes**	32 (15.9)	169 (84.1)	2.29 (1.27, 4.11)**
	**No**	14 (7.0)	187 (93.0)	
		**30-Day unplanned readmission**		
		**Yes**	**No**	
**SUN consult**	**Yes**	8 (4.0)	194 (96.0)	0.36 (0.17, 0.80)*
	**No**	22 (10.9)	180 (89.1)	

**p < 0.01, *p < 0.05

## DISCUSSION

Implementation of substance use navigation using the CA Bridge model of care with a focus on connections to low-barrier ongoing treatment and harm reduction is associated with a twofold increase in MOUD initiation and a 64% reduction in risk of 30-day unplanned readmission for hospitalized patients with AUD and/or OUD.^15,24^ These results are remarkable given that Marshall is a nonacademic, community-based hospital with no addiction medicine consult service. The implementation of substance use navigation is Marshall’s primary hospital-wide intervention for addressing SUDs. Though receipt of MOUD or MAUD prior to hospitalization was not measured as part of this study, SUN consults are primarily generated for new initiations or re-initiations of addiction treatment.

Findings from this analysis are consistent with other studies showing the utility of using patient navigation to initiate patients on MOUD/MAUD and connect them to outpatient treatment. An evaluation of a similar model of patient navigation for hospitalized patients with infections resulting from injection of substances observed both an increase in MOUD initiation and a decrease in 90-day readmissions.^25^ Other studies have provided evidence for the utility of substance use navigation in specific groups including pregnant and incarcerated people and patients presenting to the emergency department.^9,26^ A recent study by Ober et al. found that a hospital-based addiction consult service increases the initiation of MOUD in the inpatient setting. ^27^

The strong associations observed in this study highlight the effectiveness of using a trauma-informed, whole-person care model of patient navigation. Rather than relying on a treatment model based on “disease-focused, transaction-based” interactions that primarily prescribe medications, SUNs provide a touch point for patients during their hospitalization and at the time of discharge. ^28^ Growing evidence suggests that using a relationship-based health promotion and recovery model for healthcare delivery is cost-effective and improves patient care in a variety of settings, including treatment of SUDs.^9,28,29^

It is also notable that COPD and heart failure were among the top 10 primary diagnoses at discharge for patients in this study, as they are included in the list of conditions monitored by the Hospital Readmissions Reduction Program.^18^ Additionally, the most common diagnosis among patients in this study was sepsis, which is a severe condition that is costly for hospitals to treat and the subject of many quality improvement efforts.^3,30–32^ Providing whole-person, patient navigation for patients with SUDs could be an important strategy to improve quality of care, as measured by fewer unplanned readmissions and by better overall health following discharge.

### Limitations

This study may be limited by measurement error, as inclusion criteria for this study were based on ICD-10 codes associated with AUD and OUD; it is well-established that substance use disorders are underdiagnosed and under-documented in medical records, even when it is the primary reason for the hospitalization.^14,30^ Additionally, MOUD and MAUD were defined as FDA-approved medications, and there are many non-FDA approved medications (particularly for AUD) to address craving and decrease heavy drinking days that were not included in this study. Further, some patients may have been hospitalized at hospitals other than Marshall, though the next closest hospital is 23 miles away and CMS HRRP data suggests that 99% of Marshall patients who are readmitted are readmitted to Marshall.

The results of this study are also affected by the SUN’s inability to see all patients with AUD and OUD, having a better chance of seeing patients who had long lengths of stay in the hospital, and prioritizing those with the most severe AUD and/or OUD. These patients may also be more likely to initiate MOUD and MAUD and have a higher risk of 30-day unplanned readmission, making our results most generalizable to patients with more severe AUD and OUD. Additionally, this study was conducted at a single rural hospital with access to the highest quality training and resources (i.e., the CA Bridge program) for implementing an evidence-based model to address OUD and AUD in hospitalized patients, all of which limit the generalizability of these findings.

## CONCLUSIONS

Findings from this study contribute to the growing evidence that implementation of hospital-based interventions to treat SUDs can not only improve quality of care for patients but also benefit the hospital by reducing 30-day unplanned readmissions in a group that is known to be at particularly high risk for this outcome.^33–35^ The use of propensity-score matching to account for confounding factors, the retrospective cohort design, and the setting where 99% of readmissions are to this same hospital make the results of this study especially compelling.

Future research should focus on setting standards for the provision of care for hospitalized patients with SUDs, estimating the cost-effectiveness of patient navigation for SUD treatment, determining the combined effect of patient navigation and medication on patient outcomes, and identifying the impact of substance use navigation on longer term outcomes related to engagement in SUD treatment and utilization of healthcare services. Marshall’s program may serve as a model for all hospitals — but especially rural, community-based hospitals without an addiction medicine consult service — that want to use low-barrier, evidence-based interventions to improve outcomes for patients with SUDs.  

## Data Availability

De-identified study data and analytic code will be made upon request following publication and ending three years following article publication to researchers by request to the corresponding author and at the discretion of the research team.
